# Vitamin D Effects on Cell Differentiation and Stemness in Cancer

**DOI:** 10.3390/cancers12092413

**Published:** 2020-08-25

**Authors:** Asunción Fernández-Barral, Pilar Bustamante-Madrid, Gemma Ferrer-Mayorga, Antonio Barbáchano, María Jesús Larriba, Alberto Muñoz

**Affiliations:** Instituto de Investigaciones Biomédicas “Alberto Sols”, Consejo Superior de Investigaciones Científicas, Universidad Autónoma de Madrid, Instituto de Investigación Hospital Universitario La Paz, and CIBERONC, Arturo Duperier 4, 28029 Madrid, Spain; afbarral@iib.uam.es (A.F.-B.); pbustamante@iib.uam.es (P.B.-M.); gferrer@iib.uam.es (G.F.-M.); abarbachano@iib.uam.es (A.B.); mjlarriba@iib.uam.es (M.J.L.)

**Keywords:** vitamin D, cell differentiation, stemness, cancer, carcinoma cells, cancer-associated fibroblasts, cancer stem cells, organoids, epithelial-mesenchymal transition, Wnt/β-catenin

## Abstract

Vitamin D_3_ is the precursor of 1α,25-dihydroxyvitamin D_3_ (1,25(OH)_2_D_3_), a pleiotropic hormone that is a major regulator of the human genome. 1,25(OH)_2_D_3_ modulates the phenotype and physiology of many cell types by controlling the expression of hundreds of genes in a tissue- and cell-specific fashion. Vitamin D deficiency is common among cancer patients and numerous studies have reported that 1,25(OH)_2_D_3_ promotes the differentiation of a wide panel of cultured carcinoma cells, frequently associated with a reduction in cell proliferation and survival. A major mechanism of this action is inhibition of the epithelial–mesenchymal transition, which in turn is largely based on antagonism of the Wnt/β-catenin, TGF-β and EGF signaling pathways. In addition, 1,25(OH)_2_D_3_ controls the gene expression profile and phenotype of cancer-associated fibroblasts (CAFs), which are important players in the tumorigenic process. Moreover, recent data suggest a regulatory role of 1,25(OH)_2_D_3_ in the biology of normal and cancer stem cells (CSCs). Here, we revise the current knowledge of the molecular and genetic basis of the regulation by 1,25(OH)_2_D_3_ of the differentiation and stemness of human carcinoma cells, CAFs and CSCs. These effects support a homeostatic non-cytotoxic anticancer action of 1,25(OH)_2_D_3_ based on reprogramming of the phenotype of several cell types.

## 1. Introduction

Vitamin D_3_, or cholecalciferol, is a secosteroid molecule synthesized in the human skin by the action of solar UV-B light on 7-dehydrocholesterol. A second, limited source (around 10%) of vitamin D_3_ in the human organism is the diet, particularly fatty fish such as salmon, herring and sardines. Cholecalciferol is biologically inert. It enters the bloodstream and is hydroxylated at position C-25 in the liver by cytochrome CYP2R1 to render 25-hydroxyvitamin D_3_ (25(OH)D_3_, calcidiol or calcifediol). 25(OH)D_3_ is then subjected to another hydroxylation at position C-1 by cytochrome CYP27B1, mainly by kidney tubular cells and also by several types of epithelial and immune cells, to form 1α,25-dihydroxyvitamin D_3_ (1,25(OH)_2_D_3_, calcitriol) [1,2,3]. Both 25(OH)D_3_ and 1,25(OH)_2_D_3_ bind to the vitamin D receptor (VDR) protein, one of the 48 members of the ligand-activated transcription factor superfamily of nuclear hormone receptors. Although some controversy exists, 1,25(OH)_2_D_3_ appears to bind VDR with higher affinity and efficacy in terms of gene regulation than 25(OH)D_3._ Ligand binding promotes the formation of heterodimers between VDR and RXR, the receptor for 9-cis-retinoic acid, and the binding of these VDR-RXR heterodimers to DNA [1,2,3]. 

Chromatin immunoprecipitation sequencing assays have revealed the presence of around ten thousand VDR DNA binding sites in the human genome [4]. They are distributed throughout the whole genome. A subset is located close to the transcription initiation site of the genes that are directly regulated by 1,25(OH)_2_D_3_, but in many cases they are located upstream of the transcription initiation site or in introns, in the coding sequence, or downstream target genes. In line with the high number of VDR DNA binding sites, 1,25(OH)_2_D_3_ regulates the expression of hundreds of genes that vary between tissues, cell types and context [5,6]. Accordingly, 1,25(OH)_2_D_3_ displays a whole set of functions in the organism. The long-term knowledge of the association of vitamin D deficiency with rickets in children and osteomalacia in adults led to the consideration of calcium and phosphate metabolism and bone biology as the main roles of vitamin D in humans. However, during evolution, VDR was probably involved first in energy metabolism and defense against infections, and 1,25(OH)_2_D_3_ is today considered an important multifaceted regulator of the immune system [7].

1,25(OH)_2_D_3_ was initially linked to cancer in 1981, when the groups of D. Feldman and T. Suda reported its effect of inhibiting the proliferation of cultured human melanoma cells and inducing the differentiation of mouse myeloid leukemic cells, respectively [8,9]. During the last four decades, many studies have confirmed that 1,25(OH)_2_D_3_ attenuates the proliferation rate of many types of cancer cells, usually in association with a differentiation-inducing effect. In recent years, it has been shown that 1,25(OH)_2_D_3_ regulates the gene expression profile and phenotype of stromal fibroblasts present in the tumor microenvironment. Moreover, several studies have indicated that 1,25(OH)_2_D_3_ modulates cell stemness in a few cancer systems. Here, we review this set of actions, positing 1,25(OH)_2_D_3_ as a crucial regulator of homeostasis in the organism and as a candidate for non-cytotoxic anticancer therapies based on the regulation of cancer cell differentiation and stemness.

## 2. Effects of 1,25(OH)_2_D_3_ on Cancer Cell Differentiation

### 2.1. Carcinomas: The Epithelial–Mesenchymal Transition 

Carcinomas are the most frequent (around 90%) type of solid cancer. They arise from epithelial cells that, in the early steps of the tumorigenic process, lose control of their proliferation and two features of their differentiated phenotype: (i) apical–basal polarity, which is the differential distribution of proteins and lipids at distinct cell surface areas that in this way are functionally diverse, and (ii) adhesiveness, which is the ability to bind strongly to neighboring epithelial cells and to the extracellular matrix (ECM) by means of a series of specialized cell adhesion structures. The loss of epithelial differentiation results from the acquisition of a cellular program called epithelial–mesenchymal transition (EMT). EMT involves drastic changes in the pattern of gene expression, triggered by a group of transcription factors (EMT-TFs: mainly SNAIL1, SNAIL2, ZEB1, ZEB2 and TWIST1) that cause repression of the epithelial phenotype and the induction of a mesenchymal state [10,11]. Thus, diverse epithelial cell surface proteins responsible for adhesiveness—including components of adherens junctions such as E-cadherin (considered a hallmark of the adhesive differentiated epithelial phenotype), tight junctions such as claudins and occludin, and desmosomes, as well as cytoskeletal components (cytokeratins), some ECM-binding integrins and polarity regulators (such as the Crumbs, Par and Scribble complexes)—are replaced by others typical of motile fibroblastic cells. These include N-cadherin, a distinct panel of integrins, vimentin and ECM-degrading metalloproteases (MMP). As a result, epithelial cells remodel their cytoskeletons, lose cell–cell and cell–ECM adhesion, change to a front–back polarity and acquire a fibroblastic-like phenotype with motility and basal membrane invasion capacities [11,12]. EMT provides tumor cells with other features of malignancy, such as stemness and diminished apoptosis, which causes resistance to cytotoxic chemo- and radiotherapies and to immunotherapy [11].

Thus, the appearance of a carcinoma involves an initial increased proliferation capacity and also the loss of the differentiated phenotype by epithelial cells that is mostly associated with the EMT program. The increased proliferation capacity gives rise to a mass of initially benign tumor cells growing at their original tissue location, whereas the loss of the differentiated phenotype gives these cells the ability to migrate (the first requirement for cancer dissemination and metastasis) and a higher survival capacity. The EMT process is activated by a variety of agents and signals that induce or activate the EMT-TFs such as transforming growth factor (TGF)-β, Wnt, Notch and ligands of several receptors with tyrosine kinase activity and cytokine receptors. These signals usually act cooperatively and in many cases are produced by stromal cells of the tumor microenvironment [11]. EMT is a reversible and usually a partial phenomenon. It does not affect all cells in a tumor and does not completely abrogate cell polarity and adhesiveness; thus, it generates intratumoral phenotypic heterogeneity. Later during tumorigenesis, EMT is reversed by the opposite process of mesenchymal–epithelial transition (MET). By restoring cell aggregation and binding to ECM, MET probably facilitates the survival of tumor cells during circulation in the bloodstream and the initial colonization of metastatic niches [10,11].

### 2.2. Effects of 1,25(OH)_2_D_3_ on the Differentiation of Carcinoma Cells

Many studies on the effects of 1,25(OH)_2_D_3_ on carcinoma cells have been performed in colon and breast cancer due to their high incidence and mortality and due to abundant reports pointing to these two neoplasias as those that are most commonly associated with vitamin D deficiency [13]. As in most cancer cell types, 1,25(OH)_2_D_3_ reduces proliferation at the cell cycle G_0_–G_1_ transition via the inhibition of retinoblastoma protein phosphorylation by cyclin/cyclin-dependent kinase (CDK) complexes, in part through the induction of CDK inhibitors and the repression of the c-*MYC* gene [1,2,3]. Usually, the inhibition of proliferation is accompanied by a reduction in cell survival due to sensitization to apoptotic stimuli, and both effects are linked to the induction of cell differentiation.

#### 2.2.1. Colon Cancer

##### 1,25(OH)_2_D_3_ Induces Epithelial Differentiation and Inhibits EMT 

**Figure 1:**
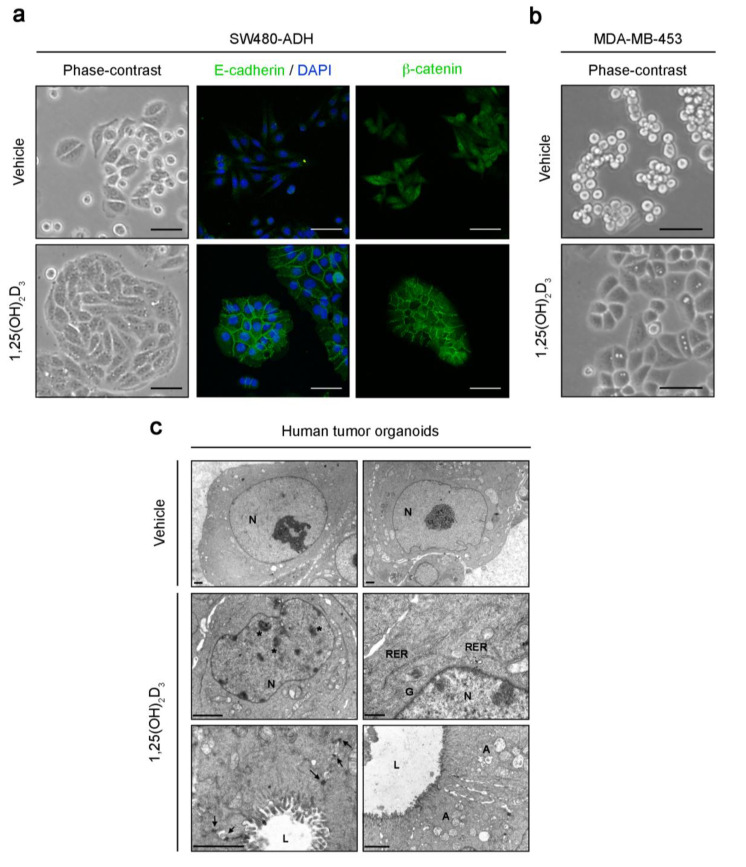
Effects of 1,25(OH)_2_D_3_ on the phenotype of human colon and breast carcinoma cells and colon tumor organoids. (**a**) Phase-contrast and immunofluorescence confocal microscopy images of SW480-ADH human colon carcinoma cells treated with 100 nM 1,25(OH)_2_D_3_ or vehicle for 72 h. Scale bars, 50 μm. (**b**) Phase-contrast microscopy images of MDA-MB-453 human breast carcinoma cells treated with 100 nM 1,25(OH)_2_D_3_ or vehicle for 72 h. Scale bars, 50 μm. (**c**) Electron microscopy images of human colon tumor organoids treated with 100 nM 1,25(OH)_2_D_3_ or vehicle for 96 h. Scale bars, 2 μm. A, autophagic vacuoles; G, Golgi complexes; L, lumen; N, nucleus; RER, rough endoplasmic reticulum; arrows, desmosomes; asterisks, heterochromatin aggregates.

**Figure 2:**
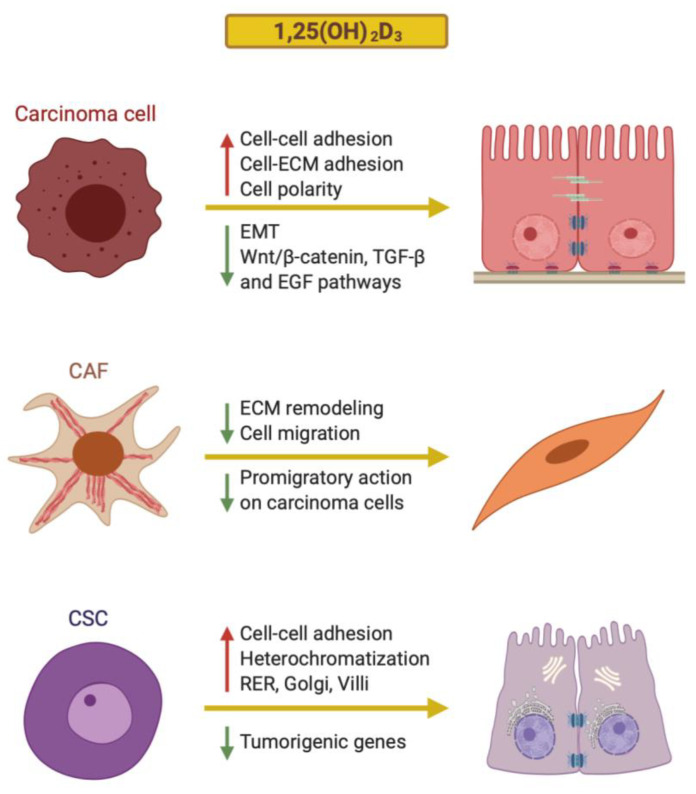
Schematic representation of the mechanisms by which 1,25(OH)_2_D_3_ regulates the differentiation of human colon carcinoma cells, cancer-associated fibroblasts (CAFs) and cancer stem cells (CSCs).

In line with its physiological role in the intestine, promoting the absorption of calcium and phosphate, the intestinal epithelium barrier function and xenobiotic metabolism, 1,25(OH)_2_D_3_ induces the differentiation of normal colon epithelial cells through the upregulation of many epithelial enzymes and markers and through maintaining the morphology typical of the epithelial differentiated phenotype [14,15]. Concordantly, in colon carcinoma cells 1,25(OH)_2_D_3_ induces a change in morphology that increases cell–cell adhesion and cell flattening (Figure 1a), which is paralleled by a decrease in proliferation. This effect is variably profound and directly related to the level of expression of VDR [16,17]. Immunofluorescence and global gene expression analyses showed that 1,25(OH)_2_D_3_ upregulates an array of intercellular adhesion molecules, including E-cadherin (Figure 1a), occludin, claudin-2 and -12, and zonula occludens/tight junction protein-1 and -2 [16,18]. 1,25(OH)_2_D_3_ induces and/or redistributes several cytokeratins, F-actin, vinculin, plectin, filamin A and paxillin, which modulate the actin cytoskeleton and the intermediate filament network, changing stress fibers and the ECM binding structures (focal adhesion contacts and hemidesmosomes) [16,17]. Thus, by controlling a large set of genes and proteins, 1,25(OH)_2_D_3_ increases cell–cell and cell–ECM adhesion (Figure 2). 1,25(OH)_2_D_3_ also induces expression of the calcium sensing receptor (CASR) that regulates calcium homeostasis and the differentiation of colon normal epithelium and carcinoma cells [19,20,21,22,23]. Curiously, 1,25(OH)_2_D_3_ has distinct effects on inhibitors of differentiation (ID)-1 and -2, two members of the ID family of proteins that control the differentiation, proliferation, migration and invasion of multiple cell types. Thus, in SW480-ADH human colon carcinoma cells, 1,25(OH)_2_D_3_ induces ID-1 but decreases ID-2 expression [24].

An in-depth study revealed that the induction of the E-cadherin protein by 1,25(OH)_2_D_3_ depends on the activation of an extranuclear signaling pathway involving the entry of Ca^2+^ from the external medium into the cytosol and the cascade activation of the RhoA small GTPase and the kinases ROCK, p38MAPK and MSK1. The activation of this pathway potentiates transcription of the *CDH1*/E-cadherin gene promoted by ligand-activated VDR, leading to the accumulation of the E-cadherin protein at the adherens junctions, along with the enhancement of cell–cell adhesion [17]. In a separate study, the kinase PIP4K2B has been reported to be necessary for E-cadherin induction by 1,25(OH)_2_D_3_ in colon carcinoma cells [25].

TGF-β is a strong inhibitor of epithelial cell proliferation and a major inducer of EMT-TFs in many cell systems. 1,25(OH)_2_D_3_ inhibits the induction of EMT by TGF-β in normal epithelial cells, which prevents the downregulation of E-cadherin [26]. Notably, TGF-β signaling is inactivated by mutation of *TGFBR2*/TGF-β receptor type II in around 30% of colon cancers or, less frequently, by that of the signal transducers *SMAD2* or *SMAD4*, which limits the EMT-promoting role of TGF-β in this neoplasia. However, Chen et al. reported that 1,25(OH)_2_D_3_ attenuates the induction of EMT by TGF-β in colon carcinoma cells and inhibits SNAIL1 and SNAIL2 expression, the E-cadherin/N-cadherin switch, and the secretion of MMP2 and MMP9 [27]. Likewise, 1,25(OH)_2_D_3_ reduces the induction of EMT by interleukin-1β via repression of the long noncoding RNA *lncTCF7* [28].

##### Antagonism of the Wnt/β -Catenin Signaling Pathway by 1,25(OH)_2_D_3_

The Wnt/β-catenin signaling pathway is abnormally activated by mutations in *APC*, or less frequently in *CTNNB1*/β-catenin or other genes, in nearly all primary human colon tumors and their metastases [29,30]. In addition, autocrine or paracrine stimulation by Wnt factors and diminished expression of Wnt inhibitors, such as members of the dickkopf (DKK) and secreted frizzled-related protein families, may potentiate this pathway [31]. As a result of these alterations, β-catenin protein accumulates within the cell nucleus of colon epithelial cells and acts as a transcriptional regulator, forming complexes with members of the T-cell factor (TCF) family and activating a specific gene expression program that largely coincides with that of normal intestinal stem cells [32]. This leads to epithelial dedifferentiation, induction of EMT and acquisition of stemness. Thus, abnormal Wnt/β-catenin pathway activation is responsible for the initiation and probably also for the progression of colon cancer via the increase in colon epithelial cell survival and proliferation and the loss of the differentiated phenotype [31,32,33].

First in colon carcinoma cells and later in other types of cancer cells, 1,25(OH)_2_D_3_ has been shown to antagonize the activation of the Wnt/β-catenin pathway at several levels [34]. Ligand-activated nuclear VDR binds to the β-catenin protein, which hampers the formation of transcriptionally competent β-catenin-TCF complexes and thus blocks the expression of their target genes [16,35]. In addition, 1,25(OH)_2_D_3_ induces *DKK-1* gene expression, which encodes an extracellular inhibitor of Wnt signaling that acts on Wnt receptor complexes at the cell surface [36]. Another mechanism of Wnt/β-catenin pathway inactivation by 1,25(OH)_2_D_3_ derives from increased accumulation of the E-cadherin protein, which, due to its high affinity, sequesters the newly synthesized cytosolic β-catenin protein at the subcortical/surface adherens junctions [16] (Figure 1a). However, this mechanism is non-essential, as 1,25(OH)_2_D_3_ inhibits the Wnt/β-catenin pathway even in cells that lack E-cadherin expression [16].

Other mechanisms of Wnt/β-catenin pathway deactivation by 1,25(OH)_2_D_3_ have been proposed: the induction of TCF4 in colon and breast carcinoma cells [37], and a paracrine action decreasing the production and release by stromal macrophages of interleukin-1β, a cytokine that inhibits the phosphorylation-mediated degradation of β-catenin protein and so increases β-catenin-TCF transcriptional activity in colon carcinoma cells [38]. In addition, in the non-malignant LT97 colon adenoma cell line, 1,25(OH)_2_D_3_ reduces the level of nuclear β-catenin and increases cellular differentiation [39].

The relevance of the vitamin D system for Wnt/β-catenin activity in colon cancer in vivo was first studied in animals. Larriba et al. [40] and Zheng et al. [41] reported that the absence of Vdr (*Vdr*-deficient mice) increases colonic tumor burden and the amount of nuclear β-catenin protein in colon cancer cells in the Apc^Min/+^ colon cancer mouse model. These findings support a role of VDR/1,25(OH)_2_D_3_ in repressing the Wnt/β-catenin pathway and the growth of intestinal tumors. More importantly, in a randomized, double-blinded, placebo-controlled clinical trial, Bostick et al. showed that supplementation with vitamin D_3_ increases the expression of E-cadherin and CASR, as well as other differentiation markers and potentially protective genes, in the healthy colon mucosa of patients with colorectal adenomas [42,43]. These authors also found an increase in the APC/β-catenin ratio in the colonic mucosa of the vitamin D_3_-supplemented group, which is compatible with an inhibitory effect of vitamin D_3_ on the Wnt/β-catenin pathway [44].

##### Other 1,25(OH)_2_D_3_ Target Genes Are Involved in Colon Cancer Cell Differentiation

Work by our group has led to the identification of several mediators of the prodifferentiation action of 1,25(OH)_2_D_3_ in colon carcinoma cells. These mediators include cystatin D, *miR-22*, KDM6B and Sprouty-2 (SPRY2).

Cystatin D is a multifunctional protein that acts as a cysteine protease inhibitor in the cytosol and extracellular region and as a transcriptional regulator within the cell nucleus [45]. The transcription of the *CST5* gene, encoding cystatin D, is strongly induced by 1,25(OH)_2_D_3_ in colon carcinoma cells via direct binding of VDR to its promoter region [46]. Remarkably, cystatin D overexpression induces the expression of E-cadherin, occludin and other adhesion proteins, and represses that of several genes encoding EMT-TFs (*SNAI1*, *SNAI2*, *ZEB1*, *ZEB2*). Moreover, cystatin D antagonizes the Wnt/β-catenin pathway and inhibits colon carcinoma cell proliferation and migration [46].

In a microarray-based study, we identified several microRNAs (miRs) regulated by 1,25(OH)_2_D_3_ in SW480-ADH human colon carcinoma cells. One of these targets is *miR-22*, which is induced by 1,25(OH)_2_D_3_ and contributes to the effects of 1,25(OH)_2_D_3_ on gene expression and cell proliferation and migration [47]. Subsequently, other groups have reported that *miR-22* inhibits EMT, invasiveness and tumor growth in colon cancer and a few other cancer systems [48,49].

KDM6B is an enzyme that demethylates di- and tri-methyl-lysine 27 on histone H3 (H3K27me2/3), an epigenetic mark that usually correlates with gene repression. Thus, KDM6B is expected to enable the activation of genes, although this histone demethylase appears to have additional transcriptional effects unrelated to histone demethylation. We found that 1,25(OH)_2_D_3_ upregulates the *KDM6B* gene by activating its promoter, and that *KDM6B* knockdown reduces the induction of E-cadherin and cell differentiation and the inhibition of β-catenin transcriptional activity promoted by 1,25(OH)_2_D_3_ in colon carcinoma cells [50]. *KDM6B* knockdown also upregulates SNAIL1 and ZEB1, the latter possibly through the decrease of *miR-200b* and *miR-200c*, and it downregulates the adhesion proteins E-cadherin, claudin-1 and -7 [50,51].

*SPRY2* was found to be a gene that is strongly repressed by 1,25(OH)_2_D_3_ in a microarray analysis performed in SW480-ADH cells [18]. Its encoded protein is a modulator of tyrosine kinase receptor signaling, with receptor- and cell type-dependent inhibitory or enhancing effects. Thus, SPRY2 inhibits fibroblast growth factor signaling but potentiates the activation of RAS-ERK by epidermal growth factor (EGF). In human colon carcinoma cells, SPRY2 promotes EMT through the upregulation of ZEB1 and the downregulation of epithelial splicing regulator ESRP1. Consequently, SPRY2 represses *CDH1*/E-cadherin and genes encoding the tight junction proteins claudin-7 and occludin, as well as the important regulators of the polarized epithelial phenotype LLGL2, PATJ and ST14 [52,53]. SPRY2 expression is induced by β-catenin in cooperation with the transcription factor FOXO3a. Accordingly, it correlates with nuclear β-catenin and FOXO3a colocalization in human colon carcinomas and is indicative of poor prognosis [54]. In summary, our data indicate that repression of *SPRY2* makes an important contribution to the prodifferentiation action of 1,25(OH)_2_D_3_.

##### Crosstalk between 1,25(OH)_2_D_3_ and EMT

The above data clearly indicate that 1,25(OH)_2_D_3_ promotes epithelial differentiation and inhibits EMT in colon carcinoma cells. Conversely, it has been shown that SNAIL1 and SNAIL2 repress VDR expression and abolish 1,25(OH)_2_D_3_ responsiveness [55,56,57]. Thus, 1,25(OH)_2_D_3_ and EMT are reciprocally downregulated and the balance between 1,25(OH)_2_D_3_ and EMT-inducing signals determines cell phenotype. Supporting this, *VDR* RNA expression correlates directly with differentiation and inversely with *SNAI1* and *SNAI2* RNA expression in human colon tumors [55,57,58,59,60]. In addition to the mechanisms described in previous sections, the mutual antagonism between 1,25(OH)_2_D_3_ and EMT is probably also a consequence of the multilevel cross-inhibition of 1,25(OH)_2_D_3_ and the EMT-inducing pathways Wnt/β-catenin, TGF-β and EGF [34,61,62] (Figure 2). For example, by repressing SPRY2 and inducing E-cadherin, which downregulates EGFR [63], 1,25(OH)_2_D_3_ may inhibit EGF signaling. In addition, cross-inhibition of VDR and EGFR has been described in colon carcinoma cells [64,65,66]. Similar functional crosstalk applies to 1,25(OH)_2_D_3_ and insulin-like growth factor (IGF)-I [61].

#### 2.2.2. Breast Cancer 

Breast cancer is a highly heterogeneous disease. Two key studies have respectively proposed five or ten molecular subtypes on the basis of a global gene expression study [67] or a more comprehensive genomic and transcriptomic analysis [68]. Analyzing the expression of estrogen receptor (ER), progesterone receptor (PR), and epidermal growth factor receptor 2 (ERBB2, NEU or HER2) is still the basis for breast cancer stratification and an important determinant of breast carcinoma cell phenotype. Cancers that lack or express very low levels of ER, PR and HER2 proteins (triple negative breast cancers or TNBC) have the poorest prognosis and, concordantly, ER^-^ PR^-^ HER2^-^ cells are highly dedifferentiated (anaplastic). Interestingly, TNBC patients have very low 25(OH)D blood levels and some in vitro and clinical studies suggest the possibility of a protective effect of 1,25(OH)_2_D_3_ against TNBC [69].

1,25(OH)_2_D_3_ contributes to the normal development and function of the mammary gland [70]. Accordingly, VDR is expressed in normal breast tissue and in many, but not all, breast carcinoma cell lines and tumors [71,72,73,74]. Several excellent reviews have summarized the effects of 1,25(OH)_2_D_3_ on breast cancer cells, animal models and patients [70,75,76,77]. The study of 1,25(OH)_2_D_3_ effects in a panel of human breast carcinoma cell lines revealed that it induces a cell-specific change in morphology (Figure 1b) and gene expression. In some cases, 1,25(OH)_2_D_3_ alters the cytoarchitecture of actin filaments and microtubules and induces cytoplasmic extensions (filopodia and lamellipodia). It also increases adhesion to substrate by promoting the accumulation of focal adhesion kinase, paxillin and α_v_- and β_5_-integrins in focal adhesion plaques. Additionally, in several cell lines 1,25(OH)_2_D_3_ upregulates E-cadherin and represses the myoepithelial proteins P-cadherin, smooth muscle α-actin and α_6_- and β_4_-integrins [72,78]. Other studies have described the inhibition by 1,25(OH)_2_D_3_ of breast carcinoma cell migration, invasion and metastatic capacities via a reduction in the expression and/or the activity of N-cadherin, the ECM components tenascin C and periostin, several metalloproteases (MMP-1, MMP-9) and serine proteases (plasminogen activator), as well as the induction of their inhibitors [79,80,81,82,83,84]. In addition, upregulation of the actin cytoskeleton adaptor protein PDLIM2 is crucial for the pro-adhesive, antimigratory and anti-invasive actions of 1,25(OH)_2_D_3_ in breast cancer cells [85]. In contrast to its induction in colon carcinoma cells [24], 1,25(OH)_2_D_3_ suppresses the expression of ID-1 in human breast carcinoma cells and xenografted tumors, which conceivably contributes to promoting differentiation [86].

#### 2.2.3. Other Solid Cancers

As in colon and breast cancers, 1,25(OH)_2_D_3_ induces cell differentiation, sensitizes to apoptosis and inhibits proliferation, migration and invasion in a series of other solid neoplasias. Again, its effects on differentiation are largely due to inhibition of the EMT and antagonism of the Wnt/β-catenin, EGF and TGF-β pathways.

The antagonism of the Wnt/β-catenin pathway by 1,25(OH)_2_D_3_ has been described in several solid cancers as a consequence of a variety of mechanisms: (1) by promoting the lysosomal degradation of LRP6, a member of the Wnt surface receptor complex, in pancreatic adenocarcinoma cells [87]; (2) via ligand-activated VDR-β-catenin interaction in renal carcinoma cells [88] and mouse skin tumorigenesis [89,90]; and (3) via ligand-activated VDR-β-catenin interaction and DKK-1 induction in Kaposi’s sarcoma cells [91]. The inhibition of the TGF-β pathway by 1,25(OH)_2_D_3_ and other VDR agonists and the subsequent repression of EMT-TFs and upregulation of epithelial markers have been reported in anaplastic thyroid cancer cells [92], ovarian cancer cells [93], lung adenocarcinoma cells [94] and renal carcinoma cells [88]. Likewise, VDR agonists repress EGF signaling in squamous cell carcinoma cells [95], epidermoid cells [96] and psoriatic keratinocytes [97].

Altogether, these effects show that 1,25(OH)_2_D_3_ is a strong, multifaceted promotor of human carcinoma cell differentiation. As discussed by Gocek and Studzinski [75], the prodifferentiation effect of 1,25(OH)_2_D_3_ on carcinoma cells does not result in the restoration of a complete normal epithelial phenotype, but it probably has an antitumor action due to the associated diminution of carcinoma cell proliferation; survival; and migratory, invasive and metastatic capacities.

### 2.3. Effects of 1,25(OH)_2_D_3_ on the Differentiation of Hematological Cancer Cells

As in solid cancers, vitamin D deficiency is common among patients with hematological malignancies, and 1,25(OH)_2_D_3_ and other VDR agonists inhibit the proliferation of leukemia, lymphoma and myeloma cells, as well as favoring their apoptosis upon cytotoxic treatments. A widely reported effect of 1,25(OH)_2_D_3_ in these cells is the inhibition of STAT-1 and STAT-3 signaling and the subsequent repression of a large number of cytokines [75,98]. However, the induction of differentiation seems to be a less important protective mechanism of 1,25(OH)_2_D_3_ in hematological malignancies than in solid cancers. Although VDR is expressed by all immune cell types, 1,25(OH)_2_D_3_ induces differentiation of myeloid leukemia cells almost exclusively [99,100]. Most results have been obtained in the acute myeloid leukemia (AML) cell lines HL60, THP-1 and U937. Thus, 1,25(OH)_2_D_3_ increases the expression of markers of the monocyte-macrophage phenotype, such as CD14 and some proteins involved in phagocytosis and adherence to the substratum, including CD11b [75,98]. A number of genes and proteins have been proposed as mediators of this prodifferentiation action of 1,25(OH)_2_D_3_, such as *CEBPB* and *CDKN1A*, which respectively encode the CCAAT enhancer binding protein β transcription factor and the p21^CIP1^ cyclin-dependent kinase inhibitor [101,102,103]. Other studies have proposed ERK, JNK, PI3K and PKC-α and -β as cytosolic mediators of these 1,25(OH)_2_D_3_ effects [104,105,106]. Notably, Muto et al. reported that 1,25(OH)_2_D_3_ induces differentiation of the retinoic acid-resistant acute promyelocytic leukemia UF-1 cell line, associated with the expression of the p21^CIP1^ and p27^KIP1^ cell cycle inhibitors [107]. Prodifferentiation effects of VDR agonists have also been reported in follicular non-Hodgkin’s lymphoma SU-DHL4 cells, with increased expression of mature B-cell markers [108].

## 3. Effects of 1,25(OH)_2_D_3_ on the Differentiation of Tumor Stromal Fibroblasts

Although the crucial importance of the tumor microenvironment during all stages of carcinogenesis is today widely accepted, studies on the action of 1,25(OH)_2_D_3_ on tumor stromal cells are scarce. Cancer-associated fibroblasts (CAFs) are the most abundant cell type in the tumor microenvironment. Recent data indicate that CAFs are a heterogeneous population of cells generated from diverse origins in response to signals secreted by tumor cells or by other cells of the tumor stroma. Thus, they can originate from the phenotypic change (activation) of resident fibroblasts, from the recruitment and activation of bone marrow-derived fibrocytes and mesenchymal stem cells, or from the transdifferentiation of other cell types (epithelial, endothelial or smooth muscle cells, adipocytes or pericytes) [109,110]. Usually, but not always, CAFs have tumor promoter effects that favor the malignancy of cancer cells by altering the ECM and secreting protumorigenic and drug-resistance factors [109,111].

Several strategies for CAF-directed anticancer therapy are possible [110]. Since CAF elimination unexpectedly rendered an acceleration of pancreatic cancer [112,113], the option of their deactivation or reprogramming to a less protumorigenic phenotype has become attractive. In this context, the VDR agonist calcipotriol has been reported to inhibit pancreatic stellate cell activation and differentiation into myofibroblasts, and thus to reduce inflammation and fibrosis in a pancreatitis mouse model and to enhance the efficacy of anticancer therapy in a pancreatic cancer model [114]. Likewise, calcipotriol reduces liver inflammation and fibrosis through the inhibition of hepatic stellate cell activation [115,116]. Accordingly, other protective effects of 1,25(OH)_2_D_3_ against fibrosis have been described and summarized elsewhere [62].

A global gene expression study, performed with human CAFs isolated from tumor biopsies of five breast cancer patients, identified 123 genes regulated by 1,25(OH)_2_D_3_ [117]. This gene signature reflects an antiproliferative and anti-inflammatory effect of 1,25(OH)_2_D_3_, as it includes the downregulation of the growth factor *NRG1* and other genes with proliferation promotion effects, as well as the upregulation of *DUSP1* (a phosphatase that inactivates MAPKs) and *NFKBIA* (an inhibitor of NFĸB). In paired normal fibroblasts (NFs), 1,25(OH)_2_D_3_ modulates the expression of 126 genes (55% of them are also regulated by 1,25(OH)_2_D_3_ in CAFs), including among the upregulated genes a few involved in antiproliferative, apoptosis and differentiation processes [117]. However, this study lacked functional analyses.

More recently, a study designed to characterize the effects of 1,25(OH)_2_D_3_ on colon cancer stromal fibroblasts rendered some interesting data [118]. First, the analysis of tumor biopsies from 658 patients showed that high VDR expression in CAFs is associated with better patient overall and progression-free survival, independently of the level of VDR expression in carcinoma cells. Second, a global gene expression analysis of seven primary cultures of CAFs and NFs established from colon cancer patient biopsies revealed that 1,25(OH)_2_D_3_ imposes in CAFs a 48-gene signature that correlates with longer patient survival in several colon cancer cohorts. Around one thousand genes are regulated by 1,25(OH)_2_D_3_ in CAFs and NFs, with a 21% overlap. The identified 1,25(OH)_2_D_3_ target genes are involved in cell adhesion, differentiation and migration; tissue remodeling; blood vessel development and inflammatory response. These genes encode mainly for ECM components and cytokines. Third, 1,25(OH)_2_D_3_ inhibits two protumoral properties in CAFs and in NFs: the paracrine promigratory action on carcinoma cells and the capacity to contract collagen gels, which is considered a hallmark of fibroblastic activation [118] (Figure 2). These results show that 1,25(OH)_2_D_3_ promotes profound reprogramming of the CAF gene expression profile, which leads to inhibition of their protumoral phenotype and contributes to protection against colon cancer. In addition, this study reveals that 1,25(OH)_2_D_3_ attenuates the malignant phenotype of colon carcinoma cells not only via a direct effect on these cells, but also indirectly through the de-activation of CAFs. Concordantly, the analysis of a large cohort of colon cancer patients indicated that the expression of VDR in CAFs and carcinoma cells has an additive protective effect, extending the overall survival of patients [118]. These findings are clinically relevant, as they indicate that colon cancer patients may benefit from an adequate vitamin D status or from treatment with VDR agonists, provided their CAFs express VDR, even though their carcinoma cells may be VDR-deficient, for instance due to SNAIL1 and/or SNAIL2 upregulation.

Remarkably, the regulation by 1,25(OH)_2_D_3_ of signaling molecules (cytokines, growth factors) secreted by CAFs suggests that it may also affect the biology of carcinoma cells and other cell types in the tumor microenvironment, such as immune and endothelial cells, in a paracrine manner. In line with these data, Kong et al. [119] reported that 1,25(OH)_2_D_3_ decreases the amount of *miR-10a-5p* found in the exosomes secreted by human pancreatic CAFs, which attenuates the promigratory and pro-invasive effects that these CAFs exert on pancreatic carcinoma cells. Notably, the possible interplay between 1,25(OH)_2_D_3_ and Wnt3A in colon fibroblasts has also been reported. Both agents are strong regulators of the gene expression profile and phenotype of these cells. However, in contrast to the antagonism reported in carcinoma cells, they have an additive and partially overlapping effect [120,121].

These results show that 1,25(OH)_2_D_3_ action extends to tumor stromal fibroblasts and that 1,25(OH)_2_D_3_ is an important regulator of CAF differentiation. Together with other findings suggesting anti-inflammatory and anti-angiogenic effects of 1,25(OH)_2_D_3_ at the level of immune and endothelial cells [122], they widen the prodifferentiation action of 1,25(OH)_2_D_3_ to cells of the tumor microenvironment, which may have important consequences on cancer development.

## 4. Effects of 1,25(OH)_2_D_3_ on Cancer Stem Cells

The concept of cancer stem cells (CSCs) states that tumors are initiated, progress and probably become resistant to therapies due to the accumulation of genetic and epigenetic alterations in tissue stem cells that become CSCs. This has led to investigations focused on identifying markers of undifferentiated stem cells in each tissue that could be used to isolate and specifically target CSCs with appropriate drugs or antibodies. However, although some relatively selective markers have been identified in a few cancer types, the idea of culturing or targeting tumor-specific CSCs has clashed with the finding of stem cell plasticity. This term refers to the dedifferentiation of cells at intermediate or even terminal differentiation stages, in order to restore the stem cell compartment following a lethal injury in normal tissues, or as a consequence of the acquisition of genetic alterations, and/or in response to signals from the tumor microenvironment in cancer [123,124,125]. Thus, stemness is today considered a usually transient cellular state that is lost in the process of differentiation. Differentiated cells can re-acquire stemness properties when the stem cell reservoir needs to be regenerated. Consequently, no stable CSCs seem to exist within tumors that can be isolated and studied as optimal targets for anticancer therapies [126].

Several studies have reported that VDR agonists inhibit the formation of floating spheroids called mammospheres (a feature attributed to CSCs) in breast cancer stem-like cells obtained from established cell lines. Interestingly, this effect could be overcome by β-catenin overexpression, which suggests that the inhibition of the Wnt/β-catenin pathway mediates this action of VDR agonists [127]. Likewise, in human triple negative and basal-like breast cancer cells, the 1,25(OH)_2_D_3_ analog BXL0124 reduces mammosphere-forming efficiency and downregulates the expression of stemness markers (*OCT4*, *CD44*) and Notch pathway genes (*NOTCH1*, *JAG1/2*) [128,129,130]. In addition, 1,25(OH)_2_D_3_ depleted the cancer stem-like cell subpopulation present in a human ovarian cancer cell line, thus reducing cell capacity to form spheres and initiate tumor formation. Additionally in this case, the main mechanism responsible for these effects of 1,25(OH)_2_D_3_ is antagonism of the Wnt/β-catenin pathway [131]. 1,25(OH)_2_D_3_ reduced sphere formation and the RNA expression of several stem cell markers (*Cd44*, *Nanog*, *Oct4*, *Sox2*, *Klf4* and *Abcg2*) in a stem-like subpopulation of mouse malignant ovarian epithelial cells [132]. Similarly, 1,25(OH)_2_D_3_ downregulates NANOG and OCT4 in embryonal carcinoma and seminoma cells [133].

The above studies share the weakness of analyzing 1,25(OH)_2_D_3_ effects on cell populations which are enriched in stem cell characteristics but which are isolated from immortalized cell lines that have been growing in culture for a long time. Organoids clearly resemble the in vivo situation more closely, as they are three-dimensional structures generated by primary normal or cancer stem cells isolated from patients on culturing. Our group has recently described the effects of 1,25(OH)_2_D_3_ on human colon normal and tumor organoids, generated from biopsies of healthy and tumor tissue obtained from colon cancer patients [134]. Remarkably, 1,25(OH)_2_D_3_ induces cell differentiation in colon tumor organoids by changing their typical blastic cell appearance to a more epithelial differentiated phenotype that includes cell–cell adhesion structures, heterochromatin, villi, abundant rough endoplasmic reticulum, Golgi complexes and autophagic vacuoles [134] (Figure 1c). Global transcriptomic RNA-seq analysis revealed that 1,25(OH)_2_D_3_ promotes an enrichment in the colon differentiation signature EPHB2^low^ vs. EPHB2^high^ in colon tumor organoids, as well as the repression of genes involved in proliferation and tumorigenesis (Figure 2). However, unexpectedly, 1,25(OH)_2_D_3_ does not substantially modulate the expression of stemness or Wnt/β-catenin target genes. Contrarily, in colon normal organoids, 1,25(OH)_2_D_3_ upregulates several key stemness genes (*LGR5*, *SMOC2*, *LRIG1*, *MSI1*, *PTK7* and *MEX3A*) and, concordantly, it does not affect the undifferentiated cell phenotype [134]. Importantly, recent RNA-seq analyses showed that 1,25(OH)_2_D_3_ has very similar effects on the global pattern of gene expression in colon and rectum normal organoids [135]. Likewise, the transcriptomic profiles induced by 1,25(OH)_2_D_3_ in colon and rectal tumor organoids are highly comparable [135]. As occurs in colon tissue [134], 1,25(OH)_2_D_3_ upregulates *LGR5*, *LRIG1*, *SMOC2* and *MSI1* stemness genes and downregulates the differentiation genes *MUC2* and *TFF2* in normal rectum organoids, but not in rectal tumor organoids, which indicates a homeostatic action of 1,25(OH)_2_D_3_ on the normal stem cell population in both intestinal areas (colon and rectum) [135]. Accordingly, studies by Augenlicht’s group have revealed that feeding mice with a low vitamin D_3_ and calcium diet or specific-inactivation of *Vdr* in Lgr5^+^ intestinal stem cells compromises stem cell properties and function, and thus alters the maturation of Lgr5^+^ progeny and intestinal homeostasis [136,137,138]. Interestingly, in human colon carcinoma cell lines, the *miR-372/373* cluster, which is upregulated by the Wnt/β-catenin pathway and enhances stemness, has been found to downregulate *VDR* RNA and a panel of differentiation genes [139]. Together, these results indicate that 1,25(OH)_2_D_3_ exerts a homeostatic action in colon normal stem cells and a prodifferentiation effect on colon CSCs.

Recently, reduced VDR expression has been found to be associated with impaired myeloid progenitor differentiation and a poor prognostic factor in AML. The observed VDR repression is mainly due to gene promoter methylation, blocking differentiation and promoting self-renewal and proliferation in myeloid precursor cells. Accordingly, VDR agonists inhibit cell stemness in normal bone marrow and AML [140].

## 5. Conclusions

The active vitamin D metabolite 1,25(OH)_2_D_3_ and other synthetic VDR agonists are differentiation agents that enforce an epithelial state in carcinoma cells largely through the induction of key epithelial proteins and the inhibition of EMT. Work done predominantly in colon and breast cancer shows that the latter is mainly a consequence of the antagonism that these molecules exert on Wnt/β-catenin, TGF-β and EGF signaling pathways. Additionally, 1,25(OH)_2_D_3_ profoundly changes the gene expression profile of tumor stromal fibroblasts. It attenuates their activated phenotype and decreases their protumoral effects. It also exerts prodifferentiation actions on CSCs. Collectively, these data indicate that 1,25(OH)_2_D_3_, or VDR agonists in general, are candidates for cancer differentiation strategies.
